# Wrap-Around Left Anterior Descending Coronary Artery Occlusion Presenting With the de Winter Pattern and Inferior ST-Segment Elevation Myocardial Infarction (STEMI): A Case Report and Comprehensive Literature Review

**DOI:** 10.7759/cureus.98496

**Published:** 2025-12-04

**Authors:** Lucio Giuseppe Granata, Francesco Amico, Marcello Marchetta, Francesca Campanella, Simona Giubilato

**Affiliations:** 1 Cardiology, Cannizzaro Hospital, Catania, ITA; 2 Cardiology, Policlinico Tor Vergata, Rome, ITA

**Keywords:** acute coronary syndrome, coronary angiography, de winter pattern, electrocardiography, heart failure with reduced ejection fraction, myocardial infarction, percutaneous coronary intervention, stemi, st-segment elevation, wrap-around left anterior descending artery

## Abstract

The left anterior descending (LAD) coronary artery originates from the left main coronary artery and runs along the interventricular groove, terminating before the apex of the left ventricle. A “wrap-around” LAD is a normal anatomical variant in which the distal portion of the vessel continues beyond the apex to supply part of the inferior wall. Its acute occlusion can frequently produce characteristic electrocardiographic changes, with ST-segment elevation in both the anterior and inferior leads. These diffuse repolarization abnormalities can make it difficult to identify the culprit artery and may even be mistaken for non-ischemic alterations, thus delaying appropriate treatment.

We report a case of wrap-around LAD occlusion presenting with unusual and distinctive ECG findings. A man in his 60s with multiple cardiovascular risk factors and recurrent myocardial infarction presented with acute chest pain. The ECG showed ST-segment elevation myocardial infarction (STEMI) in the inferior leads, associated with a de Winter pattern in the anterior leads, characterized by 1-3 mm upsloping ST-segment depression at the J point that continued into tall, symmetrical T-waves, indicative of proximal LAD occlusion or severe obstruction. Coronary angiography revealed both proximal and distal subocclusions of a wrap-around LAD, successfully treated with angioplasty without procedural complications; However, despite the good angiographic result and an optimal first medical contact-to-balloon time, the patient subsequently developed an infarction-related complication, manifesting as ischemic heart failure with mildly reduced ejection fraction (HFmrEF).

This case represents a unique demonstration of how this LAD variant can produce the simultaneous coexistence of a de Winter pattern and inferior ST-segment elevation. Although rare, the ECG signs of wrap-around LAD occlusion are distinctive and must be promptly recognized, as immediate reperfusion is indicated to prevent poor prognosis, extensive myocardial damage, heart failure, and death. In our case, despite early and successful revascularization, the patient developed ischemic HFmrEF, underscoring the severity of the consequences associated with wrap-around LAD occlusion and highlighting the potentially serious damage that may result from delayed or missed diagnosis.

## Introduction

The left anterior descending (LAD) coronary artery is typically 10-13 cm long, originates from the left main coronary artery, and courses through the interventricular groove, usually terminating before reaching the apex of the left ventricle. Along its course, it gives rise to septal perforators and diagonal branches that supply the anterior and anterolateral walls of the left ventricle, two-thirds of the interventricular septum, and the apex [1]. The LAD is commonly described in terms of proximal, mid, and distal segments. A more detailed anatomical subdivision identifies four distinct segments: the first (proximal) segment extends from the LAD origin to the first septal branch; the second spans the course between the first and third septal branches; the third runs from the third septal branch to the cardiac apex; and the fourth segment continues beyond the apex along the distal portion of the vessel [2].

This vessel shows considerable variability in its distal extent and, consequently, in the amount of myocardium it perfuses. Shorter LADs perfuse a smaller territory, whereas longer LADs supply a substantially larger area [3,4]. Its length is strongly influenced by coronary dominance patterns and carries important clinical implications, particularly in the context of myocardial infarction [2-5].

Three anatomical variants of the LAD artery have been described according to the vessel length [2,6]: Type 1 (A): the LAD terminates before reaching the left ventricular (LV) apex; Type 2 (B): it reaches the apex but does not perfuse the inferoapical segment; Type 3 (C): it wraps around the LV apex, supplying the posterior apical (inferoapical) region.

In Type 3 (C), the distal LAD extends beyond the apex into the inferior interventricular groove, forming a so-called “wrap-around LAD,” a normal anatomical variant in which the vessel continues its course along the diaphragmatic surface of the left ventricle and supplies part of the apical inferior wall, perfusing at least 25% of the inferior wall [1].

The prevalence of this variant depends on the definition used and the coronary dominance pattern: it ranges from 47% in right coronary dominance (posterior descending artery (PDA) arising from the right coronary artery (RCA)) to 87% in left dominance (PDA arising from the left circumflex coronary artery (LCx)) [2]. When “wrap-around” LAD is defined as perfusing at least 25% of the inferior wall, the prevalence is 26-35% [7]. Using a more permissive definition, any LAD branch perfusing the apex, the prevalence increases to 73-78%, while in 22%-27% of cases the vessel terminates before or at the apex [4,5,7]. It has also been shown that women more frequently present with LAD type C in both dominance patterns, a finding that contrasts with earlier anatomical reports [2]. Moreover, the so-called “hyperdominance” or “super-dominance” refers to a very rare configuration that represents an extreme form of the wrap-around pattern, in which the LAD not only courses around the apex but also gives rise to the PDA. In this setting, the LAD becomes the primary source of blood flow to the posterior and inferior myocardial regions, territories normally supplied by the RCA in right coronary dominance or, in left-dominant systems, by the LCx [6].

The wraparound LAD variant occurs in approximately 0.57% of patients with anterior ST-segment elevation myocardial infarction (STEMI) and is associated with a worse prognosis compared with a non-wrap-around LAD, whereas shorter LADs, supplying a more limited myocardial territory, are generally linked to favorable outcomes [3-5,7].

From a historical perspective, as early as 1947, William Dressler described 27 cases of myocardial infarction in which the first electrocardiogram (ECG) was obtained between one and one-quarter hours and, at most, within 12 hours of symptom onset. In 18% of cases (five patients), tall T waves in the chest leads were observed without accompanying QRS abnormalities or ST-segment elevation. He also presented the ECG of a patient recorded three hours after the onset of chest pain, showing markedly tall T waves in the precordial leads (particularly V3-V6) associated with abnormal ST-segment depression. Dressler emphasized that the high T wave represented the leading diagnostic sign in the early phase of myocardial infarction [8].

In 2008, Robbert J de Winter and colleagues first described what would later be termed the “de Winter pattern,” a rare atypical electrocardiographic sign observed in approximately 2% of anterior myocardial infarctions and caused by proximal LAD occlusion. It is characterized by 1-3 mm of upsloping ST-segment depression at the J point in leads V1-V6, continuing into tall, positive, and symmetrical T waves, along with 1-2 mm of ST-segment elevation in lead aVR [9].

Both the prevalence reported by de Winter (30 out of 1532 patients) and the characterization of the specific culprit lesion were subsequently confirmed in 2009 in a series by Verounden et al., who identified 35 cases among 1890 patients and confirmed the same culprit vessel. This ECG pattern typically occurs in younger male patients, more frequently affected by hypercholesterolemia and with a lower prevalence of prior coronary artery disease compared with those presenting with a classic STEMI [10,11].

Although the original description presented it as a static pattern, it may instead represent an early and transient phase preceding the development of anterior ST-segment elevation rather than a distinct clinical entity. This interpretation is likely explained by the fact that in the de Winter study, the ECG was recorded very early, on average 1.5 hours after symptom onset, and coronary angiography was performed within 30-50 minutes thereafter [9,12]. It is considered a high-risk myocardial infarction pattern that, despite the absence of ST-segment elevation, reflects total occlusion or severe stenosis of the proximal LAD and therefore mandates emergency reperfusion therapy [13]. 

We describe the case of a man who experienced a myocardial infarction due to wrap-around LAD occlusion, presenting with peculiar and distinctive electrocardiographic features. This represents an emblematic and very rare presentation of a de Winter pattern associated with inferior STEMI. The patient underwent successful percutaneous coronary intervention (PCI) with drug-eluting stent (DES) implantation without procedural complications. However, despite the favorable final angiographic result and an acceptable first medical contact-to-balloon time, he developed ischemic heart failure with mildly reduced ejection fraction (HFmrEF), confirming the poorer prognosis typically observed in wrap-around LAD patients.

## Case presentation

A man in his 60s was admitted to the Cardiology Division for severe and worsening chest pain associated with cold sweating that had begun at rest several hours earlier.

On admission, he was diaphoretic but hemodynamically stable with normal vital signs (heart rate 80 bpm, blood pressure 135/85 mmHg) and oxygen saturation on room air. Clinical examination revealed normal heart sounds, no murmurs, clear lung fields, and no peripheral oedema. His past medical history included smoking, hypertension, dyslipidemia, type II diabetes mellitus, and recurrent STEMIs (most recently three years earlier), previously treated with one DES in the left circumflex artery and four DES in the LAD artery. His home medications included acetylsalicylic acid (ASA) as single antiplatelet therapy, high-intensity statin therapy (atorvastatin 40 mg), bisoprolol 1.25 mg, ramipril 5 mg, and metformin.

**Figure 1:**
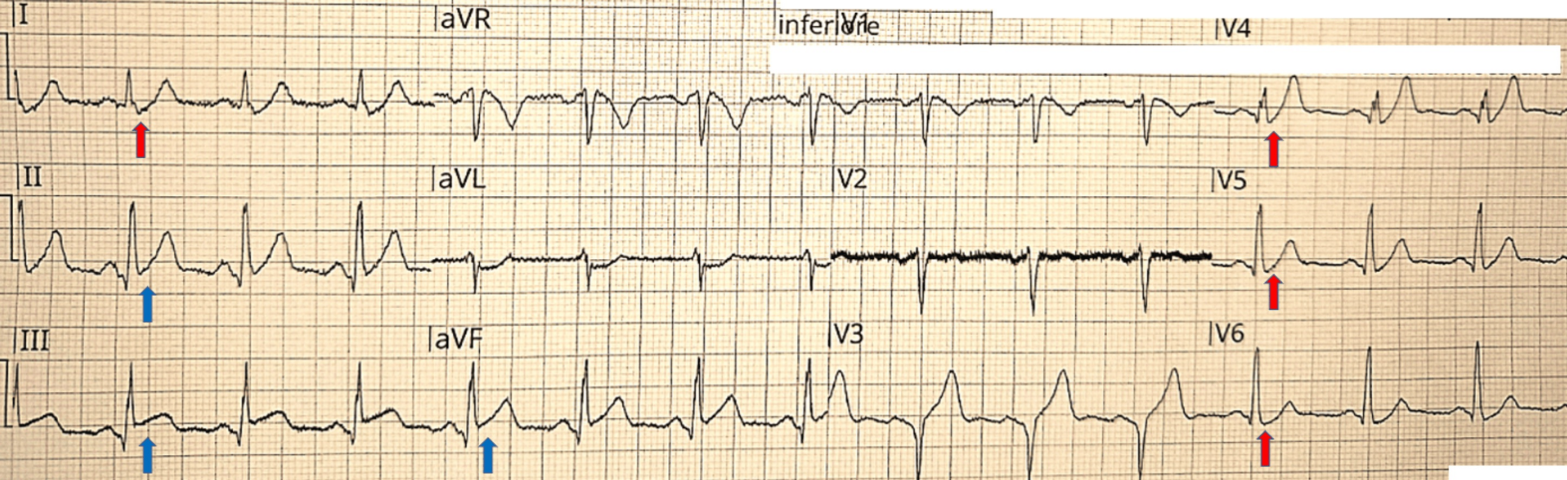
Admission ECG recorded during angina showing inferior STEMI and anterior de Winter pattern. Sinus rhythm at a heart rate of 78 bpm, with inferior ST-segment elevation (blue arrows) associated with a de Winter pattern (1 mm J-point depression with upsloping ST-segment and tall, positive T waves) in the anterior leads V4–V6 and in lead I (red arrows). Note the hyperacute T wave in lead V3. STEMI: ST-segment elevation myocardial infarction.

The initial ECG (Figure 1) showed signs of both inferior and anterior wall infarction, with ST-segment elevation in leads II, III, and aVF, associated with a de Winter pattern in leads V4-V6 (1 mm J-point depression with upsloping ST-segment and tall, positive T waves). An intravenous loading dose of ASA (300 mg) and a 5,000 IU bolus of unfractionated heparin were administered, and the catheterization laboratory was immediately activated.

**Figure 2:**
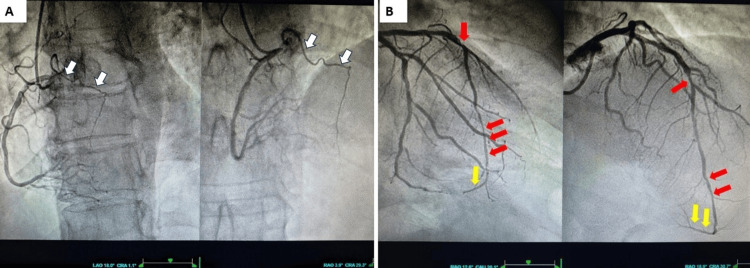
Coronary angiography. (A) LAO and RAO cranial views show a normal RCA. Note also the presence (white arrows) of a rare right coronary variant, the descending septal artery (Bonapace’s branch), arising from the right coronary sinus and supplying part of the basal interventricular septum. (B) RAO caudal and cranial views demonstrate a wrap-around left anterior descending (LAD) artery. Red arrows indicate the critical bifurcation stenosis at the distal edge of the proximal stent and the distal in-stent restenosis, while yellow arrows highlight the wrap-around course along the inferior wall. LAO: Left anterior oblique; RAO: right anterior oblique; RCA: right coronary artery.

Emergent coronary angiography surprisingly revealed a patent RCA with right dominance, along with the presence of a descending septal artery (Bonapace’s branch), a rare anatomical variant of the right coronary circulation, arising from the right coronary sinus and supplying part of the basal interventricular septum (Figure 2A, Video 1) [1]. In the left coronary circulation, a wrap-around LAD artery was identified, showing a subocclusive atherothrombotic lesion at the distal edge of the proximal stent, near the bifurcation with the diagonal branch, and a critical in-stent restenosis in the distal segment (Figure 2B, Video 2). The loading dose (180 mg) of ticagrelor was administered. 

**Video 1:** Right coronary circulation angiography. LAO caudal and RAO cranial views show a normal RCA with no evidence of stenosis and normal anterograde flow. Note also the presence of the descending septal artery (Bonapace's branch), a rare anatomical variant, arising from right coronary sinus. LAO: left anterior oblique; RAO: right anterior oblique; RCA: right coronary artery.

**Video 2:** Left coronary catheterization. RAO caudal (left panel) and RAO cranial (right panel) views show a subocclusive atherothrombotic stenosis at the distal edge of the proximal DES involving the diagonal bifurcation, as well as distal in-stent restenosis with TIMI grade 2 flow. RAO: right anterior oblique; TIMI: thrombolysis in myocardial infarction; DES: drug-eluting stent.

**Figure 3:**
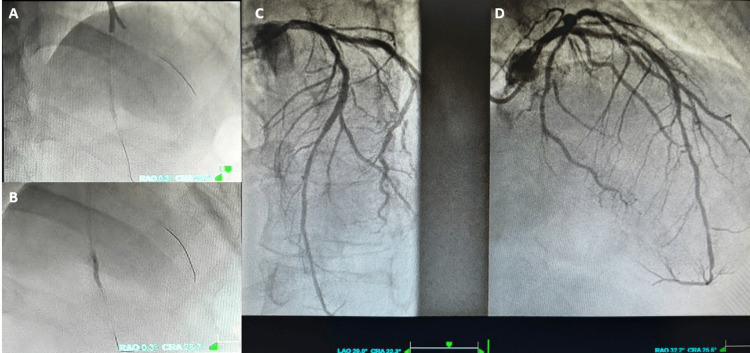
Percutaneous coronary intervention. (A) Overlapping DES implantation at the proximal LAD-diagonal branch bifurcation. (B) Balloon dilatation of distal in-stent restenosis. Final coronary angiography shows (C) LAO (D) RAO cranial views with a good angiographic result and restored distal flow (TIMI flow grade 3). DES: drug-eluting stent; LAD: left anterior descending; LAO: left anterior oblique; RAO: right anterior oblique; TIMI: thrombolysis in myocardial infarction.

PCI was performed, with implantation of overlapping DESs in the LAD bifurcation, together with multiple balloon dilatations of the distal in-stent restenosis, achieving a good final angiographic result (Figure 3). The total first medical contact-to-balloon time was approximately one hour.

**Figure 4:**
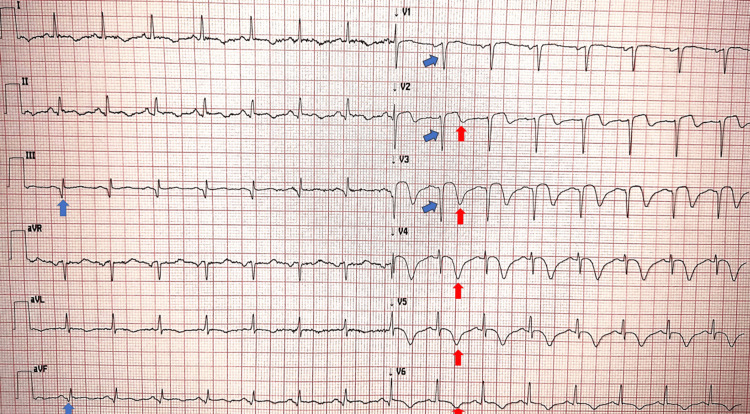
Post-PCI ECG. Red arrows show mild ST-segment elevation with biphasic T waves in leads V2–V3 and deep T-wave inversion in V4–V6, consistent with post-ischemic evolution; blue arrows show anteroseptal (“qs”) and inferior (“q”) necrosis. PCI: percutaneous coronary intervention.

The ECG obtained the following day (Figure 4) showed ventricular repolarization changes consistent with post-reperfusion ischemic evolution, with mild ST-segment elevation followed by biphasic T waves in leads V2-V3, and deep T-wave inversion in leads V4-V6. There were also anteroseptal (“qs” pattern) and inferior (leads III and aVF “q” waves) signs of necrosis.

Table 1 shows the patient’s laboratory findings during hospitalization. Hemoglobin levels, as well as renal, hepatic, and thyroid function, were within normal limits, while glucose control was suboptimal. Noteworthy laboratory values included LDL cholesterol 110 mg/dL, HbA1c 64 mmol/mol (8%), and a peak high-sensitivity troponin I level of approximately 12,000 ng/L on day 2, which subsequently declined following the typical ischemic curve, demonstrating that, despite early and optimal reperfusion, wrap-around LAD occlusion is associated with a greater extent of myocardial injury.

**Table 1:** Laboratory values during hospitalisation. WBC: white blood cell count; RBC: red blood cell count; Hb: hemoglobin; Ht: Plt: platelet count; HBA1C: hemoglobin A1C; LDH: lactate dehydrogenase; CK: creatine kinase; TG: triglyceride; HDL-C: high-density lipoprotein cholesterol; LDL-C: low-density lipoprotein cholesterol; Cre: creatinine; Na+: sodium; K+: potassium; AST: aspartate aminotransferase; ALT: alanine aminotransferase; hs-Troponin I: high-sensitivity cardiac Troponin I; CRP: C-reactive protein

Laboratory value	Day 1	Discharge	Reference range	Unit
WBC	5		4-9.5	x 10*3/microL
RBC	4.8	4.7	4-5.5	x 10*6/microL
Hb	14.5	14.4	13-17	g/dL
Plt	250	260	150-350	x 10*3/microL
hs Tn-I	500		<12	ng/L
HBA1C	64 (8)		26-46 (< 6.5)	mmol/mol (%)
Cre	0.95	1	0.6-1.1	mg/dL
Na+	138	137	135-145	mEq/L
K+	3.7	4.3	3.5-5	mEq/L
Glucose	190	98	60-100	mg/dL
Total - C	180		<200	mg/dL
HDL - C	45		40-90	mg/dL
LDL - C	110		<110	mg/dl
TG	130		<150	mg/dl
CRP	0.5	0.2	<1	mg/dL
LDH	450	180	<247	U/L
CK	280	122	<145	U/L
AST	98	55	<50	U/L
ALT	47	40	<50	U/L
Albumin	4		3.5-5.2	g/dL

Echocardiography revealed a mild reduction in left ventricular ejection fraction (EF 42%), with apical akinesia, mid-basal interventricular septal akinesia, and basal inferior wall akinesia. No significant valvular abnormalities were detected. Right ventricular systolic function was normal (TAPSE 22 mm), with mild tricuspid regurgitation, normal estimated pulmonary artery systolic pressure, a normal inferior vena cava and no pericardial effusion. The echocardiographic findings further underscored the extent of myocardial damage sustained, consistent with the dual coronary lesions involving the wrap-around LAD.

The patient was discharged after five days with normal vital signs (blood pressure 120/78 mmHg, heart rate 65 bpm), asymptomatic for angina and dyspnea. Discharge therapy included dual antiplatelet therapy (ASA 100 mg daily and ticagrelor 90 mg twice daily), with planned long-term continuation and dose de-escalation to ticagrelor 60 mg twice daily after 12 months if well tolerated. Lipid-lowering therapy was intensified with the addition of a PCSK9 inhibitor (administered subcutaneously every two weeks) to ongoing atorvastatin/ezetimibe 40/10 mg therapy. Bisoprolol 2.5 mg daily, ramipril 5 mg daily, canrenoate 25 mg daily, and combined oral therapy with gliflozin 10 mg plus metformin were also prescribed. A one-month follow-up visit was scheduled.

## Discussion

Because a wrap-around LAD supplies both the anterior wall and part of the inferior wall, its occlusion can produce simultaneous anterior and inferior ST-segment elevation, potentially obscuring the identification of the culprit artery during acute coronary syndrome (ACS) diagnosis. Although this correlation between the distribution of ST-segment elevation and the prediction of a wrap-around LAD occlusion has been described, it is not as strong or as reliable as previously assumed, since even an occlusion in the distal portion of a normally sized LAD may generate a similar pattern.

Moreover, only 20.5% of patients with occlusion involving this anatomical variant show inferior ST-segment elevation, indicating a low sensitivity for this ECG marker. However, it has been observed that in type C LAD variants, the coexistence of both anterior and inferior STEMI is more commonly seen when the occlusion occurs distal to the first diagonal branch, rather than proximally, and neither T-wave polarity nor QRS-T discordance in lead III is helpful in identifying this anatomical variant [14,15]. Furthermore, inferior ST-segment elevation may also occur in occluded type C LADs within left-dominant coronary systems [16].

Although a common anatomical variant, the wrap-around LAD has major clinical implications when involved in ACS. Its dual perfusion territory often results in a larger infarct size, more extensive myocardial injury, and more severe LV dysfunction. In anterior STEMI, this anatomy is associated with worse outcomes, including LV thrombus, stroke, stent thrombosis, ventricular arrhythmias, heart failure, and higher mortality compared with non-wrap-around LAD [3-5,7,17].

A wrap-around LAD is present in approximately 50% of patients exhibiting the de Winter pattern [9]. Nevertheless, the simultaneous occurrence of these entities is rarely reported, and concomitant inferior ST elevation is exceptionally described.

Andreou very recently described an isolated precordial de Winter phenomenon occurring in the context of total proximal in-stent occlusion involving the diagonal bifurcation of a wrap-around LAD artery. The patient was a 63-year-old hypertensive man with a history of prior PCI to both the LAD and RCA who presented with chest pain. The initial ECG was particularly remarkable, showing sinus tachycardia at approximately 100 bpm, a left anterior fascicular block, and a de Winter pattern in leads V2-V6, while leads V1 and aVR exhibited significant ST-segment elevation of 2 mm. The patient was correctly and promptly recognized and successfully treated with emergency PCI, with an overall favorable reported outcome. Although his left ventricular ejection fraction during hospitalization was 35%, it improved to the lower limits of normal (50%) at the four-month follow-up. The author underscored the importance of recognizing this ECG pattern as a STEMI equivalent, as patients may otherwise progress to a transmural infarction if not managed promptly and appropriately with primary angioplasty [18]. Unlike our case, his patient did not show inferior ST-segment elevation, supporting the concept that a more distal occlusion of the vessel is more frequently associated with concomitant inferior STEMI [14].

Previously, Tomcsányi and Littmann described two patients with acute subtotal occlusion of a proximal, large wrap-around LAD presenting with a de Winter pattern in leads V4-V6, isoelectric ST segments, and tall hyperacute T waves in V3, accompanied by ST elevation in aVR. Both cases showed anteroseptal ST elevation (V1-V3) with inferior ST depression; thus, unlike our case, inferior ST elevation was absent. In their first patient, despite early revascularization, the post-PCI ECG resembled ours-QS complexes in V1-V3, followed by positive-negative ST/T morphology and isolated T-wave inversions in V4-V6-accompanied by markedly elevated troponin. The second case demonstrated severe LV dysfunction and recurrent ventricular fibrillation, confirming the high incidence of ventricular arrhythmias and heart failure in this population. The coexistence of the de Winter pattern and anterior ST elevation within the same ECG has been described by authors as the “precordial ST-segment continuum”, reinforcing its STEMI-equivalent nature and the need for immediate percutaneous reperfusion [19].

Recently, Zheng illustrated the same phenomenon of the “ST-segment continuum in the precordial leads,” similar to that described by Tomcsányi and Littmann, but with an important novel feature. By reporting the case of a 45-year-old hypertensive and diabetic man who presented to the emergency department with continuous chest pain since the previous day, he described the unusual variant of the de Winter pattern (characterized by concomitant precordial ST-segment elevation and a de Winter sign) associated with an acute inferoposterior STEMI. Indeed, the 18-lead ECG showed ST-segment elevation in the inferior leads (II, III, aVF) and in V5-V9, initially suggesting an occlusion of the RCA or LCx. Coronary angiography, however, revealed left coronary dominance with a small, non-dominant RCA and a wrap-around LAD occlusion distal to the first septal and diagonal branches. His case highlighted the diagnostic challenge of accurately localizing the culprit artery in STEMI patients presenting with a concomitant de Winter sign [8].

Finally, the coexistence of the classic de Winter pattern and inferior STEMI in a wrap-around LAD occlusion in the setting of hyperdominance (PDA arising from LAD) was reported by Wang and Shen. They described a young hypertensive smoker presenting with chest pain and a mild elevation in troponin levels. Coronary angiography revealed a total proximal occlusion of a type C LAD. The patient underwent PCI with stent implantation, achieving a favorable clinical outcome. In this case as well, the post-procedural ECG recorded after 12 hours closely resembled ours, showing inferior "q" waves and reperfusion-related T-wave inversion in leads V2-V6; however, important differences included dominance and an isolated, complete proximal LAD occlusion [20].

Based on these observations, our case represents the first reported instance, under right coronary dominance, of concurrent inferior STEMI and anterior de Winter pattern due to wrap-around LAD occlusion with double critical stenosis: one proximal (bifurcation lesion) and one distal (in-stent restenosis). This exceptional presentation reflects the broad perfusion territory of the LAD and the complex ECG manifestations that can arise from this variant.

Despite early PCI and angiographically successful reperfusion, he developed HFmrEF. This supports prior evidence that wrap-around LAD occlusion results in substantial myocardial loss and adverse outcomes, even with timely revascularization [4,7,17].

Our case highlights the importance of early ECG recognition of atypical infarction patterns (STEMI-equivalent) and rapid intervention to minimize myocardial damage and improve prognosis. Immediate percutaneous reperfusion remains essential to prevent heart failure and mortality [9,13,18].

## Conclusions

Wrap-around LAD occlusion represents an anatomical variant capable of producing atypical and diagnostically challenging ECG patterns. This case illustrates the coexistence of de Winter morphology with inferior ST-segment elevation, a very rare association and, to our knowledge, previously unreported in right coronary dominance with underlying double critical LAD stenosis. Such presentations may hinder accurate culprit vessel identification, particularly given the frequent underrecognition of de Winter patterns.

Despite timely and successful revascularization, the patient developed HFmrEF, underscoring the substantial myocardial vulnerability associated with this anatomy. This report highlights the need for heightened diagnostic vigilance and the use of imaging and biomarkers to support early recognition and prompt revascularization in presentations that do not display classic ST-segment elevation.
